# ‘Loche’ Squash (*Cucurbita moschata*): Phytochemical Profile and Bioaccessibility of Carotenoids, Tocopherols, and (poly)Phenols

**DOI:** 10.1007/s11130-026-01551-8

**Published:** 2026-07-18

**Authors:** Felipe Jiménez-Aspee, Marilú Roxana Soto-Vasquez, Jan Frank, Guillermo Schmeda-Hirschmann

**Affiliations:** 1 Department of Food Biofunctionality (140b), Institute of Nutritional Sciences, University of Hohenheim, Stuttgart, 70599 Germany; 2 Laboratorio de Farmacognosia, Facultad de Farmacia y Bioquímica, Universidad Nacional de Trujillo, Av. Juan Pablo II s/n, Trujillo, 13011 Perú; 3 Laboratorio de Química de Productos Naturales, Instituto de Química de Recursos Naturales, Universidad de Talca, Campus Lircay, Talca, 3480094 Chile

**Keywords:** Cucurbits, *in vitro* digestion, Glutathione, Peruvian squash landrace, Intestinal Epithelial Cells, Food Processing

## Abstract

‘Loche’ squash (*Cucurbita moschata*) is an ancient cucurbit landrace from northern Peru, with deep cultural roots in the Moche cuisine tradition. To our knowledge, this is the first study to combine phytochemical profiling of raw and cooked pulp with standardized in vitro gastrointestinal digestion and intestinal cell-based assessment in this matrix. This study assessed the carotenoid, tocopherol, and (poly)phenol composition of raw and boiled pulp, evaluated bioaccessibility through standardized in vitro gastrointestinal digestion (INFOGEST), and investigated the effects of the bioaccessible fraction on intracellular glutathione levels in a Caco-2:HT29-MTX-E12 co-culture model. UHPLC-DAD-MS² tentatively identified 16 phytochemicals, including phenolic and hydroxycinnamic acids, coumarin, and glycosylated derivatives. Lutein and α-tocopherol were the predominant carotenoid and tocopherol in raw pulp, respectively. Boiling substantially reduced all quantified phytochemicals, with tocopherols showing the greatest losses (77–93%) and phenolic acids being nearly depleted (retention 7–11%). Bioaccessibility was compound-specific: zeaxanthin showed the highest micellarization efficiency (70.9%), tocopherols were moderately to highly bioaccessible, and phenolic acids were poorly recovered (≤ 3.6%). No significant effects on cell viability or intracellular glutathione levels were observed. These findings support the nutritional valorization of this culturally significant Andean crop.

**Supplementary Information:**

The online version contains supplementary material available at https://doi.org/10.1007/s11130-026-01551-8 .

## Introduction

Cucurbits, including the common squash *Cucurbita pepo*, *C. maxima*, and *C. moschata* are relevant crops with a long tradition of use in Latin American cultures. *Cucurbita* species are recognized as important ancient crops of the Americas, whose bioactive compounds, including carotenoids, cucurbitacins, phytosterols, and (poly)phenols, contribute to their nutritional and health-promoting properties [1]. The ‘Loche’ squash is a landrace of *Cucurbita moschata* Duchesne ex Poir. developed by pre-Columbian cultures along the northern Peruvian coast, whose occurrence and diversity in the southern Amazon basin of Peru has been recently documented [2] (Figure S1). The chemistry of edible *Cucurbita* species includes cucurbitosides [3], cucurbitacins, diterpenes and triterpenes [4, 5], phenolic acids [6] and lignans [5], among others, with most research efforts directed to seed bioactives and carotenoids from the pulp [7–9].

However, data on the bioaccessibility of (poly)phenols and tocopherols from *C. moschata* landraces remain absent, and changes in phytochemical composition upon cooking are poorly characterized. To our knowledge, no previous study has combined phytochemical profiling of raw and processed pulp with INFOGEST-based bioaccessibility assessment and intestinal cell assays in any *C. moschata* landrace. Therefore, this study aimed to assess the composition of carotenoids, tocopherols, and (poly)phenols in the raw and boiled ‘Loche’ squash pulp, evaluate the bioaccessibility of the main identified compounds through standardized in vitro gastrointestinal digestion (INFOGEST), and investigate the effects of the bioaccessible fraction on reduced glutathione (GSH) levels in intestinal epithelial cells.

## Materials and Methods

This section is provided as Supplementary Material.

## Results and Discussion

### Carotenoid Content, Composition and Bioaccessibility

The ‘Loche’ squash was characterized by the presence of α-carotene, β-carotene, β-cryptoxanthin, lutein, lycopene and zeaxanthin (Table 1, Figure S2), with lutein as the predominant carotenoid in pulp (2.0 ± 0.2 mg/100 g dw). Our results are consistent with reported carotenoid profiles for *Cucurbita* species [10, 11]. Carotenoid concentrations depend largely on variety, ripening stage, and geographical origin [12]. Total carotenoid contents as low as 0.61 mg/100 g have been reported for *C. maxima* pulp [13], underscoring the wide compositional variability across growing conditions and populations. Lutein has been reported in the puree of *C. moschata*, variety ‘Menina Brasileira’ at a significantly lower concentration (0.06 mg/100 g) [14], while in *C. maxima* variety ‘Exposição’, lutein content reached 1.0 mg/100 g [14], which is closer to the values reported here.

**Table 1:** Carotenoid, tocopherol, and phenolic acids content of raw and boiled ‘Loche’ squash *(Cucurbita moschata*) pulp, and concentration of the main compounds after standardized in vitro digestion

	Raw (mg/100 g)	Boiled (mg/100 g)	Digested (nmol/mL)
Carotenoids			
α-carotene	0.108 ± 0.006^a^	0.063 ± 0.030^a^	0.028 ± 0.003
β-carotene	0.208 ± 0.023^a^	0.055 ± 0.007^b^	0.089 ± 0.001
β-cryptoxanthin	0.026 ± 0.001^a^	0.005 ± 0.001^b^	0.008 ± 0.001
Lutein	1.995 ± 0.181^a^	1.110 ± 0.313^b^	0.199 ± 0.014
Lycopene	0.019 ± 0.001	ND	ND
Zeaxanthin	0.451 ± 0.027^a^	0.253 ± 0.045^b^	0.351 ± 0.040
Tocopherols			
α-tocopherol	2.346 ± 0.327^a^	0.274 ± 0.098^b^	0.635 ± 0.041
β-tocopherol	0.403 ± 0.023^a^	0.027 ± 0.001^b^	0.038 ± 0.001
γ-tocopherol	1.628 ± 0.101^a^	0.365 ± 0.031^b^	0.364 ± 0.002
δ-tocopherol	0.227 ± 0.007^a^	0.042 ± 0.004^b^	0.032 ± 0.004
Phenolic acids			
Protocatechuic acid	1147.4 ± 68.4^a^	80.3 ± 1.1^b^	0.560 ± 0.054
Caffeic acid	37.6 ± 0.5^a^	2.7 ± 0.5^b^	-
Coumaric acid	116.0 ± 14.2^a^	12.9 ± 1.1^b^	1.89 ± 0.07
Ferulic acid	19.4 ± 0.1^a^	1.76 ± 0.01^b^	-
Salicylic acid	52.7 ± 7.9^a^	4.1 ± 0.8^b^	-

Different superscript letters within the same row indicate significant differences between raw and boiled samples (*p* < 0.05, unpaired *t*-test with Welch’s correction, *n* = 3). *ND*, not detected

Boiling significantly reduced the content of all quantified carotenoids, with losses ranging from 41.6% (α-carotene) to 80.7% (β-cryptoxanthin) (Table 1). Lycopene was not detected in boiled samples, likely due to its initially low concentration. These differential losses observed among individual carotenoids can be attributed to differences in their chemical structure, chromoplast localization and polarity. β-cryptoxanthin, a monohydroxy xanthophyl, contains a hydroxyl group that increases its polarity relative to hydrocarbon carotenes, promoting leaching into the aqueous cooking medium and increasing susceptibility to heat-induced degradation [15]. In contrast, α-carotene, a non-polar hydrocarbon carotene, is more tightly associated with lipid droplets and chromoplast membranes, conferring greater resistance to aqueous leaching [15], in agreement with its statistically non-significant reduction upon boiling (Table 1). Similarly, lutein and zeaxanthin, both dihydroxy xanthophylls, showed intermediate losses (44.4 and 43.9%, respectively), while β-carotene, despite being a hydrocarbon carotene, showed a pronounced loss (73.6%), possibly reflecting its higher initial abundance and greater exposure during cell wall disruption. Overall, these losses reflect the combined effects of thermal degradation, cis-trans isomerization, and leaching of carotenoid-protein complexes into the cooking water [12, 16], although heat-induced disruption of chromoplast membranes can enhance extractability in some cultivars [17], explaining the contradictory results reported in the literature [10, 12, 14, 18].

**Fig. 1:**
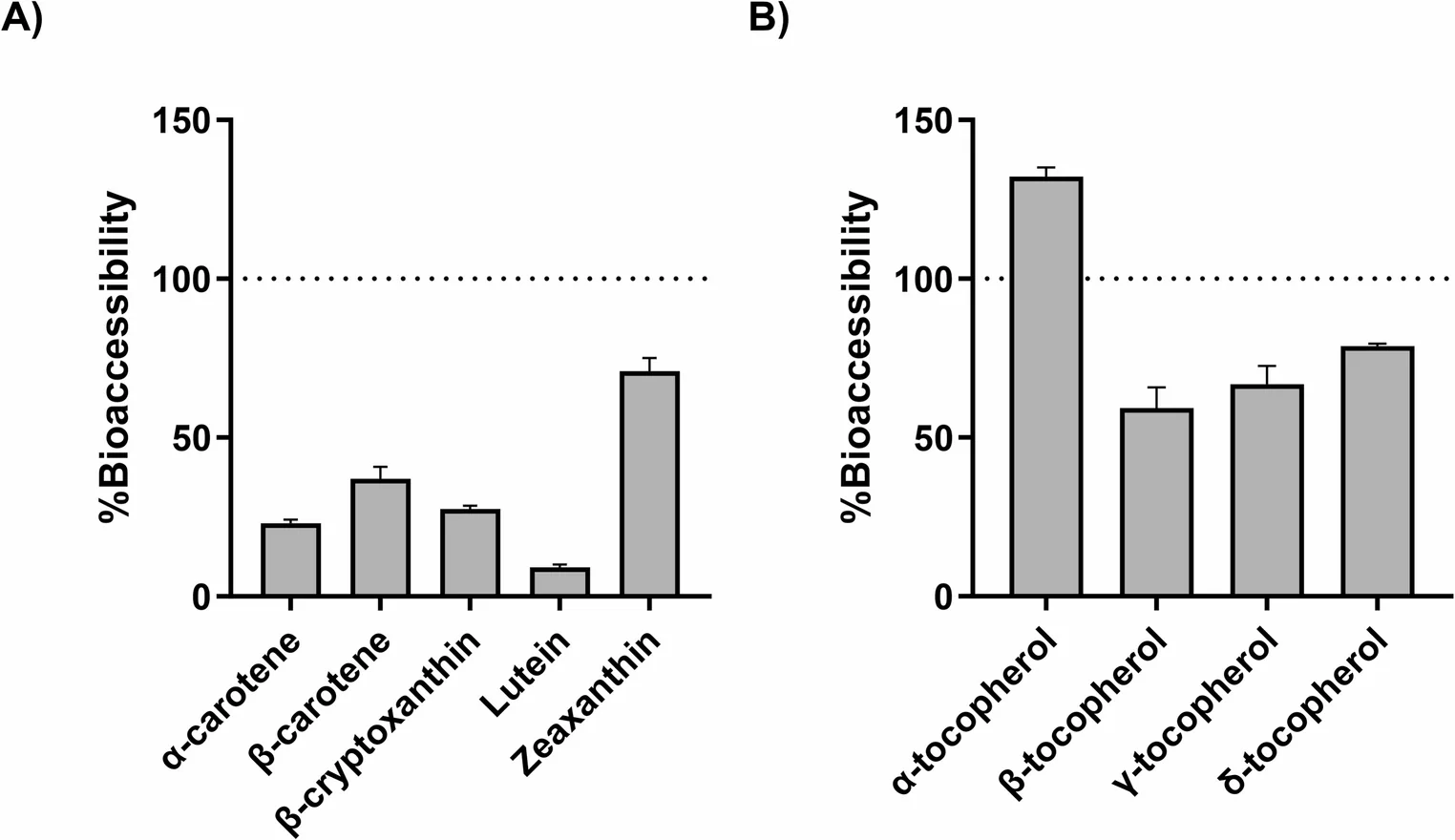
Bioaccessibility (%) of carotenoids (**A**) and tocopherols (**B**) from boiled ‘Loche’ squash (*Cucurbita moschata*) after standardized *in vitro* gastrointestinal digestion

Following in vitro digestion, bioaccessibility was strongly compound-specific, ranging from 9.2 ± 0.8% for lutein to 71 ± 4% for zeaxanthin (Fig. 1; Table 1). The pattern of lutein showing lower bioaccessibility than β-carotene regardless of its matrix concentration has been consistently reported for *Cucurbita* species and attributed to food matrix-related factors rather than substrate availability [19]. The contrasting bioaccessibility of these dihydroxy xanthophylls likely reflects their chromoplast storage form: zeaxanthin occurs in liquid-crystalline chromoplast that facilitates liberation during digestion, while lutein is stored as protein-complexes in thylakoids, limiting its release [20]. The low lipid content of squash pulp may further limit micelle formation, since dietary fat drives carotenoid solubilization [21], while dietary fiber may restrict micellarization by sequestering bile salts [22].

β-carotene (37.0%), α-carotene (23.0%), and β-cryptoxanthin (27.5%) bioaccessibility values are consistent with *Cucurbita* species reports. Lutein bioaccessibility of 10.0–13.4% and β-carotene of 25.8–35.3% [19], and β-carotene of 17–21% in *C. moschata* Butternut squash [22] have been reported. The higher β-carotene bioaccessibility here reported may reflect differences in chromoplast structure and matrix composition between landraces. β-cryptoxanthin showed intermediate bioaccessibility, consistent with its monohydroxy character, intermediate in polarity between carotenes and dihydroxy xanthophylls. These values fall within the broader range reported for carotenoid-rich vegetables: 18–20% for α-carotene, 7–49% for β-carotene, and 9–59% for lutein [23].

The relatively high bioaccessibility of zeaxanthin and β-carotene is nutritionally relevant. Zeaxanthin supports macular health [20], while β-carotene is a provitamin A precursor, suggesting that ‘Loche’ squash may contribute meaningfully to dietary intake of both micronutrients. This compound-specific variability underscores the need to consider food matrix effects when assessing the nutritional contribution of carotenoid-rich vegetables.

## Tocopherol Content, Composition and Bioaccessibility

All four Tocopherol isoforms, namely α-, β-, δ-, and γ-tocopherol, were detected in ‘Loche’ squash pulp, while no tocotrienols were found (Table 1). The predominant isoforms in raw pulp were α- and γ-tocopherol (2.35 and 1.63 mg/100 g dw, respectively), consistent with reported values for *Cucurbita* flesh [24, 25]. Notably, β- and δ-tocopherol, typically absent in *Cucurbita* species [25], were also quantified here (Table 1), possibly reflecting the unique genetic background and pedoclimatic conditions of this Peruvian landrace.

Boiling led to pronounced reductions in all isoforms, ranging from 77.5% (γ-tocopherol) to 93.3% (β-tocopherol) (Table 1**)**, likely reflecting the heat sensitivity of tocopherols and limited structural protection of the squash matrix, compounded by tissue softening and oxidative exposure of released lipids [26].

Following in vitro digestion, all tocopherol isoforms showed moderate to high bioaccessibility, ranging from 59.3% (β-tocopherol) to 132.2% (α-tocopherol) (Fig. 1, Table 1). The α-tocopherol bioaccessibility of 132.2% likely reflects underestimation of true tocopherol content in the solid matrix by saponification-based extraction, relative to efficient micellarization promoted by bile salts during digestion, a phenomenon previously reported for δ-tocopherol in beans (up to 124%) [27]. Lipids intrinsic to the squash matrix and derived from digestive secretions likely supported solubilization into mixed micelles [28], yielding bioaccessibility values exceeding those reported for other *Cucurbita*-based matrices [29] and comparable to those described for beans [27]. These findings highlight the nutritional relevance of ‘Loche’ squash as a dietary source of bioaccessible vitamin E.

## Phytochemical Characterization and Bioaccessibility of (poly)Phenols in Raw, Boiled, and Digested ‘Loche’ Squash

The UPLC-DAD-MS^2^ analysis of squash samples allowed the tentative identification of 16 compounds, including organic acids, phenolic acids, hydroxycinnamic acids, coumarin, and glycosylated derivatives (Table 2, Figure S3). The profile was dominated by low molecular weight phenolic acids, more abundant in the raw sample and partially reduced after boiling, consistent with previous surveys of *Cucurbita* matrices [25, 30]. Full MS^2^ annotation details are provided in Table 2.

**Table 2:** Phytochemical compounds tentatively identified in raw, boiled and in vitro digested ‘Loche’ squash (*Cucurbita moschata*) pulp by UPLC-DAD-MS^2^

#	RT (min)	Molecular formula	Mode (+/-)	m/z (amu)	Error (ppm)	MS^2^ products	UV Max	Tentative identification	Raw	Boiled	Digested
1	2.31	C_6_H_7_O_7_	-	191.0192	0.611	111.0077	216	Citric acid	X	X	X
2	3.45	C_4_H_5_O_4_	-	117.0184	0.155	73.0295	214	Succinic acid	X	X	-
3	4.36	C_7_H_5_O_5_	-	169.0137	0.580	125.0234	271	Gallic acid	X	-	-
4	4.44	C_10_H_11_O_7_	-	243.0510	1.491	125.0234	270	Galloyl glycerol	X	-	-
5	5.69	C_7_H_5_O_4_	-	153.0187	0.475	109.0284	271	Protocatechuic acid	X	X	X
6	6.39	C_9_H_9_O_4_	-	181.0503	0.745	135.0442	272	Dimethoxybenzoic acid	X	-	-
7	6.65	C_7_H_5_O_3_	-	137.0235	0.179	93.03333	300	Salicylic acid	X	X	-
8	7.09	C_7_H_11_O_5_	-	175.0606	0.570	115.0390	-	Isopropyl malic acid	X	X	-
9	7.36	C_18_H_25_O_10_	-	401.1453	1.037	269.1030, 161.0446	254	Benzyl beta-primeveroside	X	X	X
10	7.85	C_9_H_7_O_4_	-	179.0344	0.555	135.0443	320	Caffeic acid	X	X	-
11	8.74	C_12_H_15_O_4_	-	223.0978	1.335	179.1071	300	Resorcinolic acid isomer	X	-	-
12	8.88	C_9_H_7_O_2_	+	147.0442	0.214	91.0549	310	Coumarin	X	X	-
13	9.15	C_18_H_27_O_10_	-	403.1613	1.477	223.0974, 179.1071	310	Resorcinolic acid hexoside	X	X	-
14	9.19	C_9_H_7_O_3_	-	163.0392	0.279	119.0492	320	*p*-Coumaric acid	X	X	X
15	11.32	C_10_H_9_O_4_	-	193.0502	0.655	134.0363	320	Ferulic acid	X	X	-

Among the identified compounds, gallic acid (3) and protocatechuic acid (5) were confirmed as benzoic acid-type phenolics by authentic standards, while three hydroxycinnamic acids, namely caffeic acid (10), *p*-coumaric acid (14) and ferulic acid (15), were similarly confirmed and showed relatively higher thermal stability than gallate-related phenolics. The organic acid fraction (citric acid, succinic acid, isopropyl malic acid) was relatively stable under boiling conditions. Coumarin (12) and salicylic acid (7) persisted after boiling. The presence of coumarin is subject to regulatory limits in certain food categories (EU Regulation 1334/2008), though quantitative assessment would be required to evaluate its relevance here.

Benzyl-β-primeveroside (9) was one of the few metabolites detected after in vitro digestion, suggesting its dissacharide-conjugated structure confers gastrointestinal stability. Compounds 11 and 13 were tentatively annotated as resorcinolic acid (C_12_H_16_O_4_) and its hexoside, respectively, based on accurate mass and MS^2^ fragmentation. Sinapic acid was excluded based on the high mas error (37.420 ppm), despite previous reports of its occurrence in some *Cucurbita* cultivars [31].

Quantitatively, the phenolic profile was dominated by protocatechuic acid, followed by *p*-coumaric acid (Table 1), in contrast to *C. moschata* Butternut squash, where syringic acid dominates [22], highlighting compositional variability across landraces. Boiling nearly depleted all phenolic acids, with retention of only 7–11% of the initial content, consistent with thermal degradation and leaching into the cooking medium [30]. After digestion, only protocatechuic acid and *p*-coumaric acid were quantifiable, with very low bioaccessibility (0.17 ± 0.02% and 3.61 ± 0.07%, respectively). Caffeic, ferulic, and salicylic acid were not detected in the bioaccessible fraction (Figure S4). Caffeic acid is known to undergo extensive chemical transformation during gastric digestion (oxidation, dehydration, esterification), which may explain its absence [32]. Ferulic acid is frequently esterified or bound to cell-wall polysaccharides, requiring enzymatic hydrolysis prior to release. This bound fraction often remains insoluble and undetected in plant-derived foods, consistent with previous reports for *C. moschata* [22].

The higher bioaccessibility of *p*-coumaric acid relative to protocatechuic acid suggests that structural class, rather than hydroxylation pattern alone, drives digestive persistence. Hydroxycinnamic acids are generally more readily absorbed in free form, whereas hydroxybenzoic acids form stronger matrix associations [32]. Nonetheless, both compounds showed very low overall bioaccessibility, indicating that most phenolic acids in ‘Loche’ squash likely reach the colon intact, consistent with reports that up to two-thirds of ingested hydroxycinnamic acids escape upper gastrointestinal absorption reaching the colon [33], where they may become available for biotransformation into smaller phenolic catabolites.

Glycosylation alone did not guarantee stability: benzyl β-primeveroside (9) persisted while the resorcinolic acid hexoside (13) did not, indicating that aglycone structure and conjugation type jointly determine gastrointestinal resistance. Overall, bioaccessibility was governed by polarity, substitution pattern, structural class, and conjugation type rather than initial abundance in the boiled matrix.

## Cell Viability and Antioxidant Effects

The complete bioaccessible fraction obtained after INFOGEST digestion of boiled ‘Loche’ squash, containing carotenoids, tocopherols and phenolic acids, was standardized by total phenolic content (TPC, Folin–Ciocalteu) to allow consistent dosing across independent replicates, yielding consistent values of 255–271 µg GAE/mL. Cells were exposed to 5, 25, and 50 µg GAE/mL alongside a digestion blank. LDH release remained below 4% cytotoxicity at all concentrations after 24 h (Table S1), confirming cellular safety and supporting the use of 50 µg GAE/mL as the maximum concentration for subsequent assays, consistent with recommendations for coupling in vitro digestion with epithelial cell models [34].

Intracellular GSH levels were evaluated in differentiated Caco-2:HT29-MTX-E12 co-cultures after 24 h exposure. Results were normalized to untreated controls due to inter-experimental variability in basal GSH between passages. N-acetylcysteine (750 µmol/L) significantly increased intracellular GSH levels (*p* < 0.01), confirming assay responsiveness (Figure S5). Treatment with the bioaccessible fraction at all tested concentrations did not significantly modify basal GSH levels, though a modest non-significant increase was observed at 50 µg GAE/mL. The digestion blank showed no significant effects.

The absence of a GSH-modulating effect is consistent with the very low phenolic acid bioaccessibility observed, limiting achievable intracellular concentrations. Additionally, differentiated intestinal epithelial cells maintain robust endogenous antioxidant defenses, including millimolar GSH concentrations and multiple GSH-dependent enzymatic systems [35], which may attenuate detectability of modest effects under non-stressed conditions. Future studies using oxidatively stressed cell models may reveal the cytoprotective potential of the bioaccessible fraction.

## Conclusion

This study provides the first integrated characterization of phytochemical composition, cooking-induced changes, and compound-specific bioaccessibility of carotenoids, tocopherols, and (poly)phenols in ‘Loche’ squash (*Cucurbita moschata*), combining UPLC-DAD-MS² profiling, INFOGEST digestion, and a Caco-2:HT29-MTX-E12 intestinal co-culture model. Three findings emerge from this work. First, lutein and α- predominated in raw pulp, and protocatechuic and *p*-coumaric acid were the main phenolic acids; notably, all four tocopherol isoforms were detected, unusual for *Cucurbita* species. Second, boiling substantially reduced all phytochemicals, with tocopherols (77–93% losses) and phenolic acids (retention 7–11%) most affected, underscoring the importance of assessing processed rather than raw forms. Third, bioaccessibility was strongly compound-specific: zeaxanthin showed the highest micellarization efficiency (71%), tocopherols were moderately to highly bioaccessible, and phenolic acids were poorly recovered (≤ 3.6%). The bioaccessible fraction did not affect GSH levels in intestinal co-cultures, consistent with low phenolic bioaccessibility and robust basal antioxidant defenses.

These findings support the nutritional valorization of ‘Loche’ squash as a source of bioaccessible carotenoids and tocopherols. Future studies should address inter-varietal variation, oxidative stress models, and colonic biotransformation of unabsorbed phenolics. Limitations include the use of a composite sample from a single market location and cell assays conducted under non-stressed conditions.

## Supplementary Information

Supplementary Material 1.

## Data Availability

The data supporting the findings of this study are available from the corresponding author upon reasonable request.

## Abbreviations

dw: Dry Weight
GAE: Gallic Acid Equivalents (mg GAE/mL)
GSH: Reduced Glutathione
INFOGEST: International Network of Food and Digestion
LDH: Lactate Dehydrogenase
ND: Not Detected
TPC: Total Phenolic Content
UPLC-DAD-MS²: Ultra-High Performance Liquid Chromatography Coupled to Diode Array Detection and Tandem Mass Spectrometry
