# Supplementary material for: ‘Loche’ Squash (Cucurbita moschata): Phytochemical Profile and Bioaccessibility of Carotenoids, Tocopherols, and (poly)Phenols
Source: Plant Foods Hum Nutr. 2026 Jul 18;81(3):98. doi: 10.1007/s11130-026-01551-8 (PMC13380564; doi:10.1007/s11130-026-01551-8)
Supplement: Supplementary file 1 — Supplementary Material 1. [file 11130_2026_1551_MOESM1_ESM.docx]

**Supplementary Material**

**'Loche' squash (*Cucurbita moschata*): phytochemical profile and bioaccessibility of carotenoids, tocopherols, and (poly)phenols**

Felipe JIMÉNEZ-ASPEE, Marilú Roxana SOTO-VASQUEZ, Jan FRANK, Guillermo SCHMEDA-HIRSCHMANN

Felipe JIMÉNEZ-ASPEE^1,*^, Marilú Roxana SOTO-VASQUEZ^2^, Jan FRANK^1^, Guillermo SCHMEDA-HIRSCHMANN^3^

*^1^Department of Food Biofunctionality (140b), Institute of Nutritional Sciences, University of Hohenheim, 70599 Stuttgart, Germany. E-mail:* [*Felipe.jimenez@nutres.de*](mailto:Felipe.jimenez@nutres.de) *(FJA);* [*jan.frank@nutres.de*](mailto:jan.frank@nutres.de) *(JF)*

*^2^ Laboratorio de Farmacognosia, Facultad de Farmacia y Bioquímica, Universidad Nacional de Trujillo, Av. Juan Pablo II s/n, 13011 Trujillo, Perú. E-mail:* [*msoto@unitru.edu.pe*](mailto:msoto@unitru.edu.pe)

*^3^ Laboratorio de Química de Productos Naturales, Instituto de Química de Recursos Naturales, Campus Lircay, Universidad de Talca, 3480094 Talca, Chile. E-mail:schmeda.hirschmann@gmail.com*

*Corresponding author: [felipe.jimenez@nutres.de](mailto:felipe.jimenez@nutres.de)

**Materials and Methods**

*Chemicals*

HPLC-grade solvents were obtained from Carl Roth (Karlsruhe, Germany). Pefabloc® was purchased from Thermo Fischer Scientific (Dreieich, Germany). Reference standards for carotenoids (lutein, zeaxanthin, β-cryptoxanthin, lycopene, α-carotene, β-carotene, and β-apo-8′-carotenal methyloxime), as well as tocochromanol quantification (α-, β-, γ-, δ-tocopherol and α-, β-, γ-, δ-tocotrienol) were purchased from Sigma-Aldrich (Taufkirchen, Germany). Folin Ciocalteu reagent, gallic acid, LDH kit (MAK529), GSH kit (MAK517), *N*-acetylcysteine, Triton X-100, DMEM high glucose, and fetal bovine serum were also from Sigma-Aldrich.

*Plant material*

The ‘Loche’ landrace of *Cucurbita moschata* Duchesne ex Poir., 1786 (n= 10 fruits) was purchased at La Hermelinda market, Trujillo, Peru, in July 2023 (Figure S1). Fruits were washed, peeled, and the seeds were removed. Pulp from all fruits was combined and homogenized to obtain a composite sample representative of the batch. Three independent one kg portions of raw composite pulp were freeze-dried using a Scanvac Coolsafe 55-15 Pro lyophilizer (Labogene, Allerød, Denmark), yielding 168 ± 22 g of raw material per replicate. In parallel, three independent one kg portions were boiled in water (1:5 w/v) for 20 min as per traditional preparation and subsequently freeze-dried affording 148 ± 13 g per replicate. Each portion was treated as an independent technical replicate for all subsequent analyses. Freeze-dried powders were stored at -30 °C protected from the light until analysis.

*Carotenoid extraction and quantification*

Carotenoids were analyzed following the procedure described by Eckhof et al. [1], with slight modifications. Freeze-dried squash samples (raw or boiled, 100 mg) and aliquots from *in vitro* digested material (1 mL) were transferred to light-protected tubes. β-Apo-8′-carotenal methyloxime (12 µL in 100 mL ethanol) was added as internal standard, followed by 0.02% BHT in ethanol as antioxidant and 50% KOH (w/v) for saponification. Samples were incubated at 70 °C for 30 min in the dark with shaking. After cooling on ice, samples were neutralized with acetic acid, and 15% NaCl (*w/v*) was added to aid phase separation. Carotenoids were extracted three times with 2 mL of hexane:diethyl ether (1:1, *v/v*) by vortexing and centrifugation (2000 × *g,* 3 min), collecting the organic phase after each extraction. The combined organic fractions were evaporated to dryness using a vacuum concentrator (RVC 2-33 CDplus, Martin Christ, Osterode am Harz, Germany).

The dried extracts were reconstituted in 200 μL of a 1:1 (*v/v*) mixture of eluent A (acetonitrile:methanol, 7:3 *v/v*) and eluent B (acetonitrile:1,4-dioxane:methanol, 37:60:3 *v/v/v*). A 20 μL injection volume was analyzed on a Shimadzu Prominence HPLC system equipped with an LC-20 AT pump, DGU-14A degasser, CTO-10AS column oven, and SPD-20A UV/Vis detector (λ = 450 nm). Chromatographic separation was achieved on a Develosil RP-Aqueous C30 column (250 × 4.6 mm, 5 μm particle size; Phenomenex, Aschaffenburg, Germany), maintained at 40 °C. The gradient program was as follows: 0 min, 100% A; 5 min, 75% A; 20, 0% A; 25 min, 0% A; 28 min, 100% A, at a flow rate of 1.5 mL/min.

For quantification, calibration curves were generated using lutein (0.05-0.5 mg/L, r² = 0.9973), zeaxanthin (0.05-0.5 mg/L, r² = 0.9913), β-cryptoxanthin (0.05-0.5 mg/L, r² = 0.9982), lycopene (0.05-0.5 mg/L, r² = 0.9643), α-carotene (0.1-1 mg/L, r² = 0.9961), and β-carotene (0.5-5 mg/L, r² = 0.9945), with the internal standard as reference. Carotenoid concentrations in solid samples are expressed as mg per 100 g of dry weight; concentrations in digesta are expressed as nmol/mL.

*Tocochromanol extraction and quantification*

Tocochromanols were quantified according to the method described by Eckhof et al. [1], with minor adaptations. Freeze-dried squash samples (100 mg, raw or boiled) and aliquots of digested squash (1 mL) were placed into light-protected tubes. KOH (600 µL, 50% *w/v*) and 1% ascorbic acid in ethanol (2 mL) were added, and samples were saponified at 70 °C for 30 min. After cooling on ice, 0.1% BHT was added (25 µL), followed by water (1 mL) and glacial acetic acid (600 µL) for neutralization. Lipid-soluble components were extracted three times with 2 mL hexane by manual inversion (1 min) and centrifugation (2000 × *g,* 3 min), collecting the upper organic phase after every extraction. The combined fractions were evaporated to dryness using a vacuum concentrator and reconstituted in 100 μL of methanol:ethanol (8:2, *v/v*).

For HPLC analysis, 10 μL of extract was injected into a Jasco system equipped with an LC-Net II/ADC controller, P-U2080 Plus pumps, AS-2059-SF Plus auto-injector, CO-2060 Plus column oven, LG-2080-02S mixer, DG-2090-53 degasser, and an FP-2020 Plus fluorescence detector (excitation 296 nm, emission 325 nm, gain 1000). Chromatographic separation was achieved on a Kinetex PFP column (150 × 4.6 mm, 2.6 μm particle size; Phenomenex), maintained at 40 °C, with a mobile phase of MeOH:H₂O (83:17, *v/v*) under isocratic conditions for 20 min at a flow rate of 1 mL/min.

Calibration curves were prepared with commercial standards of α-tocopherol (0.4-40 µM, r² = 0.9961), β-tocopherol (0.2-20 µM, r² = 0.9997), δ-tocopherol (0.2-20 µM, r² = 0.9998), and γ-tocopherol (0.2-20 µM, r² = 0.9998). Additional standards of α-, β-, γ-, and δ-tocotrienols were injected at 2 µM for retention time confirmation; however, no tocotrienol peak were detected in any sample. Results for solid samples are expressed as mg per 100 g of dry weight; concentrations in digesta are expressed as nmol/mL.

*(poly)Phenol analysis*

Extraction of phenolic compounds from raw and boiled lyophilized pulp (3 g) was performed following the procedure optimized for *Cucurbita* species [2]. Samples were mixed with EtOH:H₂O (1:1, v/v) at a solid-to-liquid ratio of 1:30 (*w/v*) and sonicated in an ultrasonic bath (35 kHz) for 10 min. The extracts were vacuum-filtered, and the process was repeated four times. The combined filtrates were evaporated to dryness and lyophilized to determine extraction yield. For enrichment of free (poly)phenols, the EtOH:H₂O extract was dissolved in water, filtered, and loaded onto an Amberlite® XAD7HP column (80 cm × 40 mm). The column was rinsed with 1 L of water to remove sugars and salts, followed by elution with 300 mL of 1% acetic acid in ethanol (*v/v*) and subsequently 100 mL of ethyl acetate. Both eluates were concentrated under reduced pressure, freeze-dried and used for UPLC-DAD-MS^2^ analysis. Aliquots of digested material (1 mL) were mixed with MeOH (1:1, *v/v*) to precipitate bile acids and salts, centrifuged (16,100 x *g*, 4 °C, 10 min), and the supernatant was directly injected without further enrichment.

Phenolic compounds from raw, boiled, and digested samples were analyzed on a Q-Exactive Plus Orbitrap mass spectrometer (Thermo Fisher Scientific, Waltham, MA, USA) coupled to an Agilent 1290 Infinity II UHPLC system equipped with a G4212 diode array detector, G4226 autosampler (maintained at 10 °C), G4220 binary pump, and G1316 column oven. Chromatographic separation was achieved on a Waters HSST3 column (2.1 × 150 mm, 1.8 μm) maintained at 40 °C. The mobile phase consisted of water with 0.2% formic acid (A) and acetonitrile with 0.2% formic acid (B), delivered at 0.3 mL/min with a 5 µL injection volume. The gradient program was as follows: 0–1 min, 99% A / 1% B; 1–12 min, linear ramp to 60% A / 40% B; 12–14 min, linear ramp to 5% A / 95% B, held until 18 min; 18–20 min, return to 99% A / 1% B; and re-equilibration until 21 min. UV absorbance was monitored from 190 to 600 nm at 2 nm interval.

Mass spectrometry was performed using a heated electrospray ionization (HESI) source operating in both positive and negative ionization modes. Source parameters were: spray voltage +4200 V / −3500 V, capillary temperature 360 °C, sheath gas 60 arb. units, auxiliary gas 20 arb. units, probe heater temperature 380 °C, and S-lens RF level 55. Full MS scans were acquired at a resolution of 70,000 (AGC target 3 × 10⁶, maximum injection time 100 ms) over *m/z* 100–1500. Data-dependent MS² fragmentation (Top5) was performed at a resolution of 17,500 (AGC target 1 × 10⁶, maximum injection time 60 ms), with an isolation window of 1.6 *m/z* and stepped normalized collision energies of 12, 30, and 60. Dynamic exclusion was set to 2.0 s (positive) and 3.0 s (negative mode). Instrument control and data analysis were performed using XCalibur version 4.0 (Thermo Fisher Scientific).

Phenolic acids, namely protocatechuic acid, caffeic acid, *p*-coumaric acid, ferulic acid, and salicylic acid, were quantified against external calibration curves. Results for solid samples are expressed as mg per 100 g dry weight; concentrations in digesta are expressed as nmol/mL.

*Simulated gastrointestinal digestion*

*In vitro* digestion was performed following the standardized INFOGEST procedure [3]. Simulated salivary, gastric, and intestinal fluids were prepared according to the guidelines of this method. A single batch of enzymes and bile salts was used throughout all experiments: α-amylase (4.4 U/mg), pepsin (787 U/mg), pancreatin (11.7 U/mg of trypsin activity), and bovine bile extract (2.83 mmol/mg bile acids), all obtained from Sigma-Aldrich. Since ‘Loche’ squash is not consumed raw, only boiled samples were subjected to *in vitro* digestion. Digestions were conducted in triplicate.

For each assay, freeze-dried boiled squash powder was weighed to 1.00 ± 0.1 g and combined with 1.6 mL of pre-heated simulated salivary fluid, 10 µL CaCl₂ (0.3 mol/L), 200 µL of α-amylase solution (final activity 75 U/mL), and 190 µL of water. This mixture was incubated for 2 min at 37 °C with shaking at 180 rpm. The gastric step was then initiated by adding 3.2 mL of gastric fluid, adjusting the pH to 3.0 ± 0.05, and supplementing with pepsin (200 µL, 2000 U/mL), CaCl₂ (2 µL), and water. Samples were maintained under the same incubation conditions for 2 h. Meanwhile, pancreatin and bile extracts were dissolved in warm intestinal fluid, vortexed, sonicated (5 min, 35 kHz), centrifuged (2000 × *g*, 4 °C), and kept on ice until use. At the completion of the gastric phase, 3.4 mL of simulated intestinal fluid was added to the chyme, the pH adjusted to 7.0 ± 0.05, and bile (1 mL) and pancreatin (2 mL, 100 U/mL) were incorporated along with CaCl_2_ (16 µL) and water to a final intestinal volume of 16 mL. Samples were flushed with nitrogen and incubated for an additional 2 h at 37 °C, 180 rpm. To terminate digestion, Pefabloc® was added at a final concentration of 1 mmol/L [4]. The digesta were briefly vortexed, centrifuged for 30 min (16,100 × *g*, 4 °C), filtered through a 0.22 µm disk filter and stored in 1.5 mL aliquots at −80 °C until further analysis.

*Bioaccessibility calculation*

The concentration of carotenoids, tocopherols and phenolic acids was determined in the soluble fraction obtained after centrifugation and filtration through a 0.22 µm membrane at the end of the *in vitro* gastrointestinal digestion, and results are expressed in nmol/mL units, reflecting the concentration of each compound in the form accessible for intestinal absorption. The percentage bioaccessibility was calculated as the nmol of each compound released from 1 g of sample into the final volume of the gastrointestinal solution divided by the total nmol present in 1 g of the undigested boiled sample, expressed as a percentage [5]. Concentrations in solid samples expressed as mg/100 g dry weight were converted to nmol using the respective molecular weights prior to bioaccessibility calculations.

*Caco-2:HT29-MTX-E12 cell culture*

Caco-2 (ECACC 09042001) and HT29-MTX-E12 (ECACC 12040401) cell lines were purchased from the European Collection of Authenticated Cell Cultures (ECACC, Salisbury, UK). Caco-2 cells were cultured in high-glucose DMEM (4500 mg/L) supplemented with 10% fetal bovine serum (FBS), 1% sodium pyruvate, 1% non-essential amino acids (NEAA), and 100 U/mL penicillin–100 µg/mL streptomycin. HT29-MTX-E12 cells were maintained in high-glucose DMEM containing 10% FBS, 1% L-glutamine, and 100 U/mL penicillin–100 µg/mL streptomycin, with sodium pyruvate and NEAA omitted. For co-culture, the medium consisted of high-glucose DMEM supplemented with 10% FBS, 1% sodium pyruvate, 1% NEAA, 1% L-glutamine, and 100 U/mL penicillin–100 µg/mL streptomycin. All cultures were maintained at 37 °C in a humidified atmosphere with 5% CO₂ (HERAcell® 150, Thermo Fisher Scientific, Langenselbold, Germany). For experimental treatments, phenol-red free high-glucose DMEM was supplemented with 2% FBS, 1% sodium pyruvate, 1% NEAA, 1% L-glutamine and 100 U/mL penicillin–100 µg/mL streptomycin.

Co-cultures of Caco-2 and HT29-MTX-E12 cells established as previously described [6]. When cells reached ~80% confluence, they were detached using 0.05% trypsin–EDTA and seeded in 48-well plates at a 90:10 ratio (Caco-2:HT29-MTX-E12). Cultures were differentiated over 21 days with medium renewal every 2–3 days. Experimental assays were performed on day 22.

*Cell viability*

The co-culture model was established in 48-well plates with Caco-2:HT29-MTX-E12 cells (ratio 90:10) at a density of 3 × 10⁵ cells/well and differentiated for 21 days. Prior to cell-based assays, *in vitro* digested squash was standardized to total phenolic content using the Folin–Ciocalteu assay and expressed as gallic acid equivalents (GAE), following the procedure described by Jiménez-Gutiérrez et al. [7]. Cytotoxicity was determined by measuring the levels of lactate dehydrogenase released into the cellular supernatant using a commercial kit (MAK529, Sigma-Aldrich). Co-cultures were exposed to: a) *in vitro* digested squash (5-50 µg GAE/mL); b) digestion blank; or c) 0.1% Triton X-100 (*v/v*) as a positive control for maximum cytotoxicity. All treatments were prepared in experimental medium. At the end of the incubation period, 50 µL of culture supernatant was collected and mixed with 80 µL of LDH detection reagent. Absorbances were measured at 500 nm after 10 min of incubation at room temperature. Cytotoxicity of the samples was calculated as follows:

$$\text{\%Cytotoxicity= }\frac{\text{A}_{\text{sample}}\text{-}\text{A}_{\text{control}}}{\text{A}_{\text{Triton X100}}\text{-}\text{A}_{\text{control}}}\text{ x 100}$$

*Effects on cellular reduced glutathione (GSH) content*

Caco-2:HT29-MTX-E12 co-cultures (ratio 90:10) were seeded in 48-well plates at a density of 3 × 10⁵ cells/well, with medium refreshed every other day. After a 21-day differentiation period, cells were treated for 24 h with *in vitro* digested squash (5-50 µg GAE/mL), blank of digestion, *N*-acetylcysteine (750 µmol/L) as a positive control, or experimental medium as basal control. At the end of the incubation, culture medium was completely removed by vacuum aspiration and cells were washed with PBS. Cells were recovered using 0.05% trypsin (10 min, 37 °C) and a cell scraper, centrifuged (1000 × *g*, 10 min, 4 °C) and then washed with cold PBS. After a second centrifugation (1000 × g, 10 min, 4 °C), cells were re-suspended with cold MES-EDTA buffer (50 mmol/L and 1 mmol/L, respectively, pH 6.3) and lysed by probe sonication (3 x 5 s pulses on ice). GSH content was determined using a colorimetric kit (MAK517, Sigma Aldrich) and results are expressed as % GSH relative to the basal control.

*Statistical analysis*

Results are presented as mean values ± standard deviation of three technical replicates. Normality was assessed using the D'Agostino–Pearson test. Differences in phytochemical content between raw and boiled samples were assessed using an unpaired t-test with Welch's correction. For comparisons among three or more groups, one-way ANOVA with Tukey's post hoc test was applied. When normality was not met, the Kruskal–Wallis test with Dunn's post hoc correction was used. All statistical analyses were performed using GraphPad Prism 10.2.3 (GraphPad Software, La Jolla, CA, USA), with p < 0.05 considered statistically significant.

**References**

[1] Eckhof P, Márquez K, Kruger J, Nina N, Ramirez-Jara E, Frank J, Jiménez-Aspee F (2024) Bioaccessibility of carotenoids, tocochromanols, and iron from common bean (Phaseolus vulgaris L.) landraces. Food Res Int 194:114935. https://doi.org/10.1016/j.foodres.2024.114935.

[2] Pinna N, Ben Abbou S, Ianni F, Angeles Flores G, Pietercelie A, Perretti G, Blasi F, Angelini P, Cossignani L (2024) Phenolic compounds from pumpkin pulp: Extraction optimization and biological properties. Food Chem X 23:101628. https://doi.org/10.1016/j.fochx.2024.101628.

[3] Brodkorb A, Egger L, Alminger M, Alvito P, Assunção R, Ballance S, Bohn T, Bourlieu-Lacanal C, Boutrou R, Carrière F, Clemente A, Corredig M, Dupont D, Dufour C, Edwards C, Golding M, Karakaya S, Kirkhus B, Le Feunteun S, Lesmes U, Macierzanka A, Mackie A, Martins C, Marze S, McClements D, Ménard O, Minekus M, Portmann R, Santos C, Souchon I, Singh R, Vegarud G, Wickham M, Weitschies W, Recio I (2019) INFOGEST static in vitro simulation of gastrointestinal food digestion. Nat Protoc 14:991–1014. https://doi.org/10.1038/s41596-018-0119-1.

[4] Kondrashina A, Arranz E, Cilla A, Faria M, Santos-Hernández M, Miralles B, Hashemi N, Rasmussen M, Young J, Barberá R, Mamone G, Tomás-Cobos L, Bastiaan-Net S, Corredig M, Giblin L (2024) Coupling in vitro food digestion with in vitro epithelial absorption; recommendations for biocompatibility. Crit Rev Food Sci Nutr 64:9618–9636. https://doi.org/10.1080/10408398.2023.2214628.

[5] Garrett D, Failla M, Sarama R (1999) Development of an in vitro digestion method to assess carotenoid bioavailability from meals. J Agric Food Chem 47:4301–4309. https://doi.org/10.1021/jf9903298.

[6] Le N, Altenburger M, Lamy E (2023) Development of an Inflammation-Triggered In Vitro "Leaky Gut" Model Using Caco-2/HT29-MTX-E12 Combined with Macrophage-like THP-1 Cells or Primary Human-Derived Macrophages. Int J Mol Sci 24: https://doi.org/10.3390/ijms24087427.

[7] Jiménez-Gutiérrez M, Zielinski C, Esquivel P, Frank J, Irías-Mata A, Jiménez-Aspee F (2025) Bioaccessibility and anti-inflammatory activity in Caco-2 cells of phytochemicals from industrial by-products of coffee (Coffea arabica L.). Journal of Functional Foods 133:107008. https://doi.org/10.1016/j.jff.2025.107008.

**Table S1.** LDH cytotoxicity (%) in Caco-2:HT29-MTX-E12 co-cultures

| **Condition** | **% Cytotoxicity (range from n =3)** |
| --- | --- |
| Digested Squash 5 µg GAE/mL | 0.4 ± 0.6 |
| Digested Squash 25 µg GAE/mL | 0.6 ± 0.8 |
| Digested Squash 50 µg GAE/mL | 1.2 ± 1.9 |
| Blank of digestion | 1.2 ± 1.6 |
| Triton X-100 | 100 ± 0.4 |
| Untreated cells | 0.0 ± 0.2 |

| **a)** |  |
| --- | --- |
| 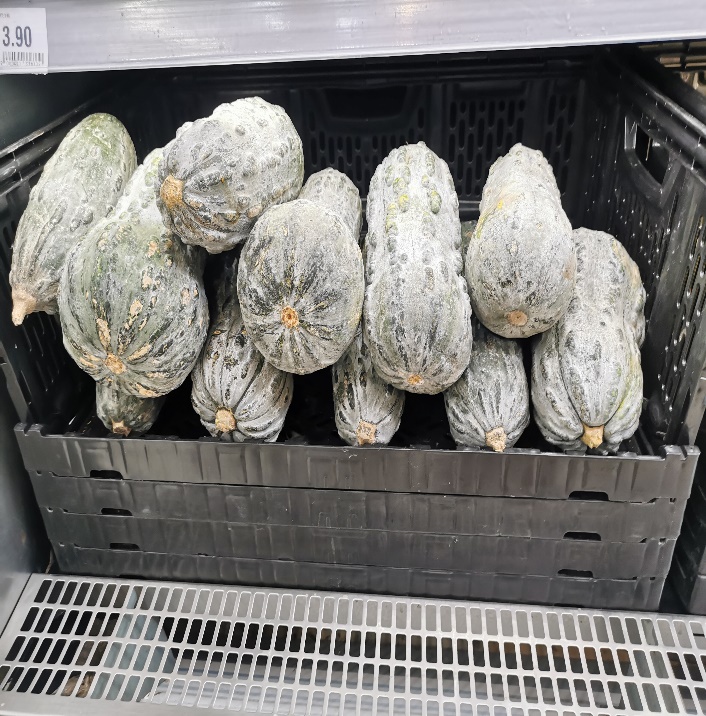 | |
| **b)** | **c)** |
| 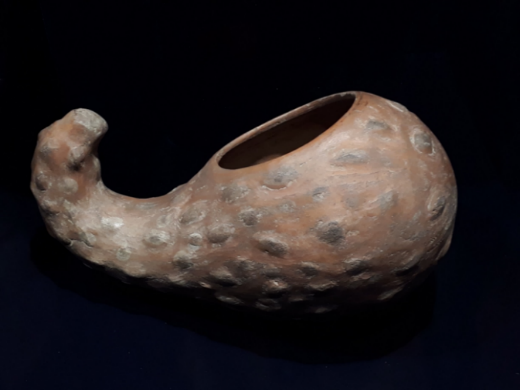 | 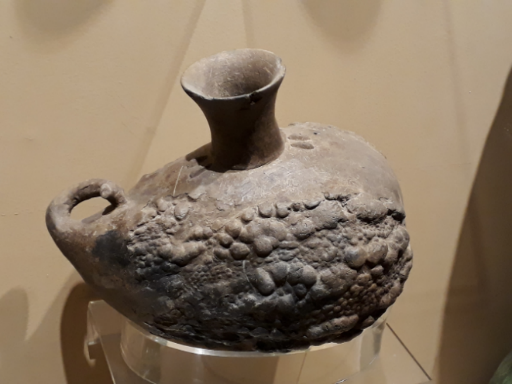 |

**Figure S1.** **The 'Loche' squash (*Cucurbita moschata* Duchesne ex Poir.) and its cultural heritage. a)** Fruits purchased at La Hermelinda market, Trujillo, Peru, used in this study. **b)** Ceramic vessel depicting loche squash from the Museo Cao, Complejo Arqueológico El Brujo, located in Magdalena de Cao, Ascope Province, La Libertad, Peru. **c)** Ceramic vessel depicting loche squash from the Huacas de Moche Museum “Santiago Uceda Castillo,” La Libertad, Peru.


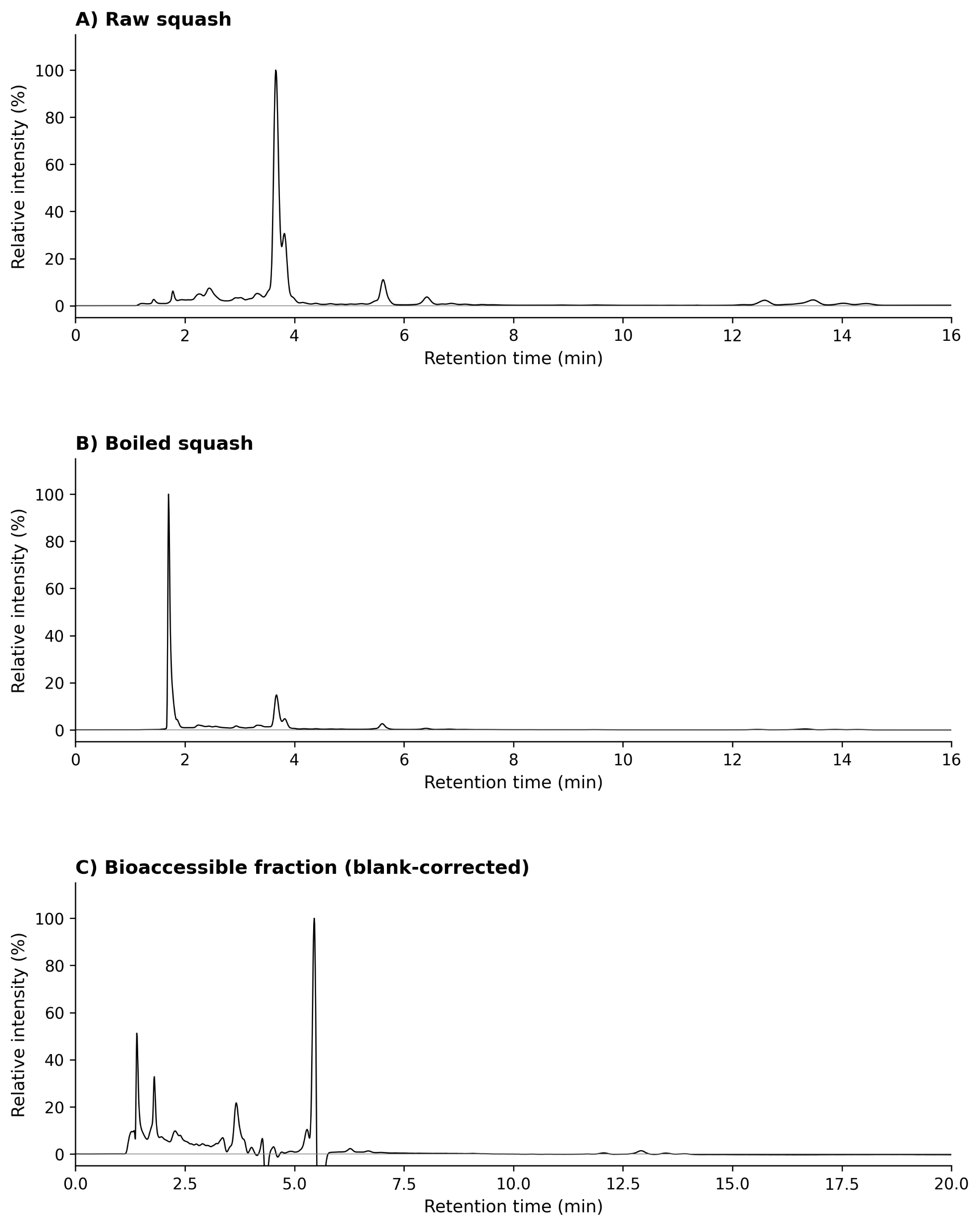


**1**

**2**

**IS**

**3**

**4**

**5**

**IS**

**3**

**1**

**2**

**4**

**5**

**IS**

**1**

**2**

**3**

**4**

**5**

**Figure S2.** Representative HPLC-UV chromatograms (450 nm) of carotenoids in **(A)** raw 'Loche' squash pulp, **(B)** boiled 'Loche' squash pulp, and **(C)** the bioaccessible fraction obtained after standardized *in vitro* gastrointestinal digestion (INFOGEST), after correction for the digestion blank. Each panel is normalized independently to its own maximum intensity. Peak identification: **1**, Lutein (3.65 min); **2**, Zeaxanthin (3.79 min); **IS**: β-apo-8′-carotenal methyloxime (5.60 min); **3**, β-Cryptoxanthin (6.38 min); **4**, α-Carotene (12.58 min); **5**, β-Carotene (13.40 min).


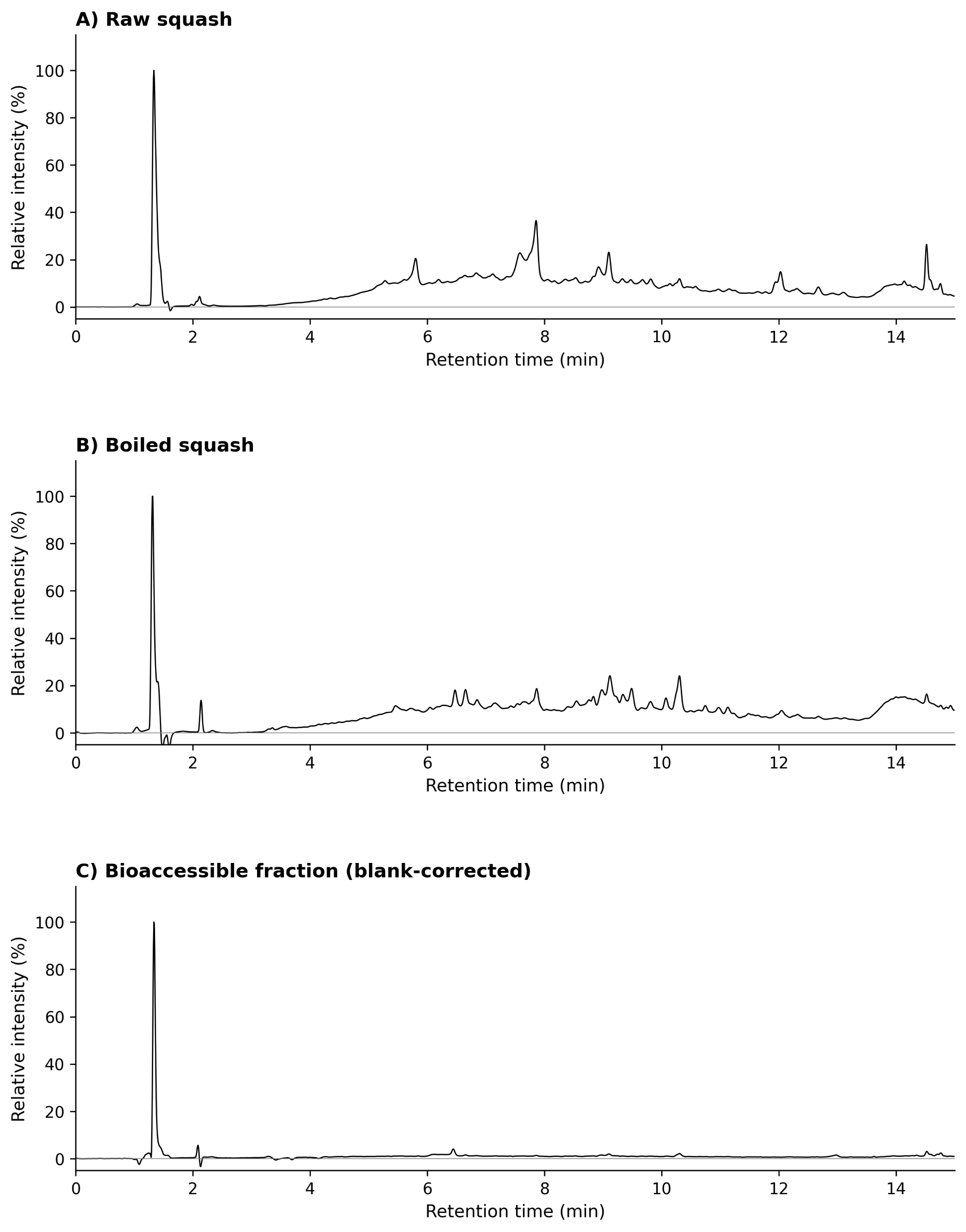


**2**

**1**

**3 4**

**5**

**6 7 8**

**9**

**10**

**11**

**12**

**13 14**

**15**

**2**

**1**

**5**

**7 8**

**9**

**10**

**12**

**13 14**

**15**

**1**

**5**

**9**

**14**

**Figure S3.** Representative UV chromatograms (300 nm) of (poly)phenolic compounds in **(A)** raw 'Loche' squash pulp, **(B)** boiled 'Loche' squash pulp, and **(C)** the bioaccessible fraction obtained after standardized *in vitro* gastrointestinal digestion (INFOGEST), after correction for the digestion blank. Each panel is normalized independently to its own maximum intensity. See Table 2 for compound identification and retention times.


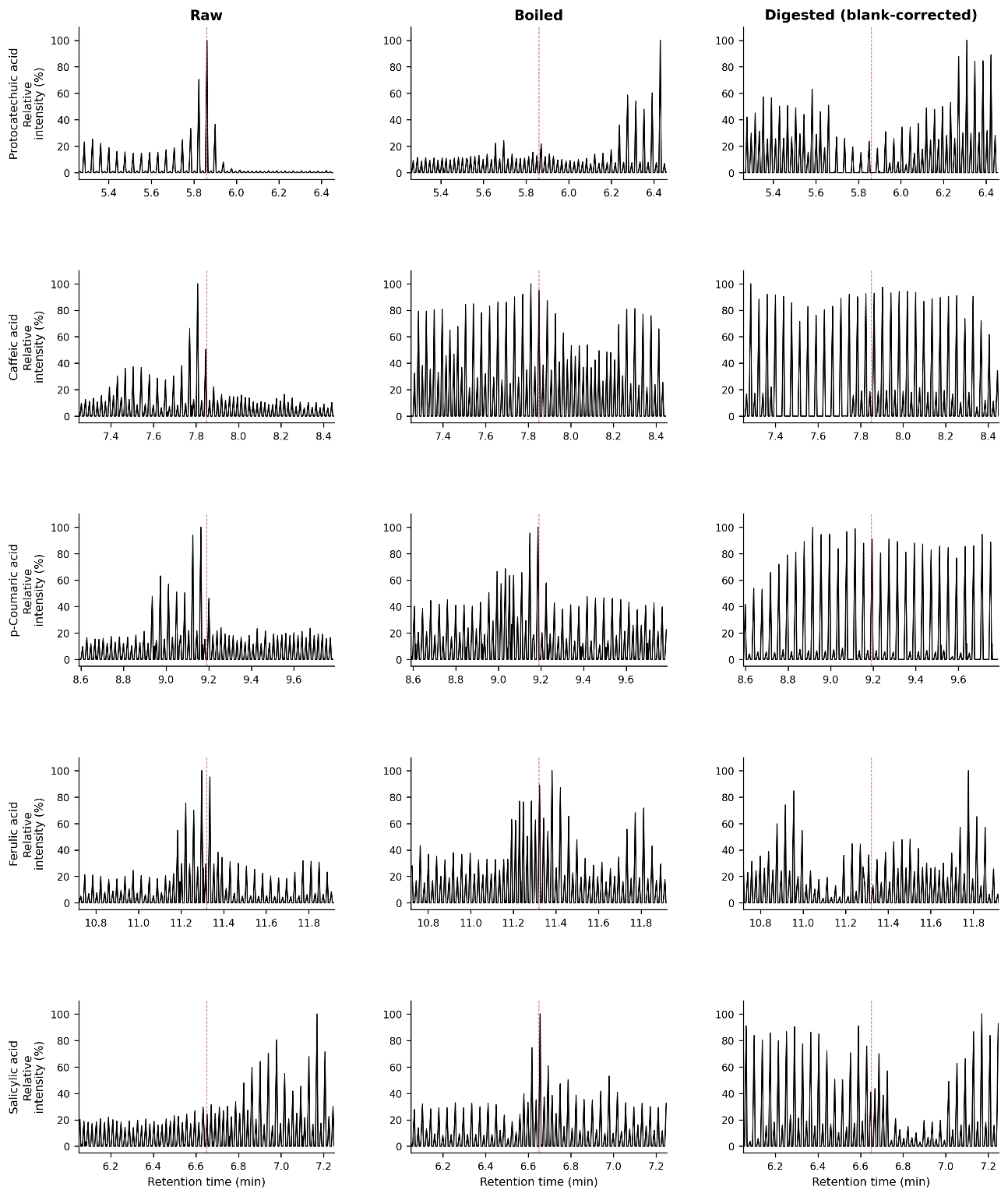


**Figure S4.** Extracted ion chromatograms (EIC) of the five quantified phenolic acids in raw, boiled, and digested (blank-corrected) 'Loche' squash samples. Rows correspond to protocatechuic acid (*m/z* 153.0187), caffeic acid (*m/z* 179.0344), *p*-coumaric acid (*m/z* 163.0392), ferulic acid (*m/z* 193.0502), and salicylic acid (*m/z* 137.0235), all in negative ionization mode. Columns show raw, boiled, and digested (after subtraction of the digestion blank) samples. Each panel is normalized independently to its own maximum intensity within a ±0.6 min window centered on the compound's retention time (red dashed line). Relative intensity in the digested panels reflects the low overall bioaccessibility of phenolic acids reported in Table 1; caffeic, ferulic, and salicylic acids show signal levels comparable to the digestion blank, consistent with their non-detection in the bioaccessible fraction.

**Figure S5.** Effects of the bioaccessible fraction of 'Loche' squash (*Cucurbita moschata*) on intracellular reduced glutathione (GSH) levels in differentiated Caco-2:HT29-MTX-E12 co-cultures. Cells were treated for 24 h with *in vitro* digested squash (5, 25, and 50 µg GAE/mL), digestion chyme blank, N-acetylcysteine (750 µmol/L) as positive control, or experimental medium as basal control. Results are expressed as % relative GSH normalized to the untreated control (dashed line). Values are mean ± standard deviation of three independent experiments. **p* < 0.01 vs. control (one-way ANOVA, Tukey's post hoc test).
